# Correlation between body mass index and gender-specific 28-day mortality in patients with sepsis: a retrospective cohort study

**DOI:** 10.3389/fmed.2024.1462637

**Published:** 2024-10-08

**Authors:** Chong Li, Huaping Huang, Qingjie Xia, Li Zhang

**Affiliations:** ^1^Department of Osteoporosis, The First People’s Hospital of Kunshan Affiliated with Jiangsu University, Kunshan, China; ^2^Department of Graduate Office, The First People’s Hospital of Kunshan Affiliated with Jiangsu University, Kunshan, China; ^3^Department of Anesthesiology, The First People’s Hospital of Kunshan Affiliated with Jiangsu University, Kunshan, China

**Keywords:** sepsis, obesity, mortality, body mass index, gender

## Abstract

**Objective:**

To investigate the potential correlation between body mass index (BMI) and the 28-day mortality rate among sepsis patients and the gender difference in this association.

**Design:**

The current research was a retrospective cohort study.

**Participants:**

A total of 14,883 male and female cohorts of sepsis patients were included in the Medical Information Mart for Intensive Care IV (MIMIC-IV V2.2) database. Patients in each gender cohort were further classified as underweight, normal weight, overweight, or obese according to BMI and the World Health Organization (WHO) BMI categories.

**Outcomes:**

The 28-day mortality from the date of ICU hospitalization was the primary outcome measure.

**Results:**

The BMI and 28-day mortality exhibited an L-shaped relationship (*p* for nonlinearity <0.001) with significant gender-specific differences. Subgroup analysis revealed different association patterns between the male and female cohorts. Specifically, BMI and mortality exhibited a U-shaped curve relationship among the males (*p* for nonlinearity <0.001) and an L-shaped relationship among the females (*p* for nonlinearity = 0.045).

**Conclusion:**

This study proposes a link between extreme BMI and 28-day mortality in patients with sepsis. Underweight patients have an increased risk of mortality; however, this risk decreases in overweight and obese patients. Upon stratifying by sex, a U-shaped pattern was observed, indicating an association between BMI and 28-day mortality in males, while an L-shaped pattern emerged in females.

## Introduction

In the last 3 years, there has been a significant rise in the worldwide occurrence of overweight and obese individuals. The overall incidence has risen from 28.8 to 36.9% among males, and among women, it has increased from 29.8 to 38.0% (1). Obesity traits are linked to the leading global causes of death (2), with discernible gender differences in the risk impact of certain diseases. For instance, research indicates that overweight women have a higher risk of type 2 diabetes than men. In comparison, overweight men have a higher risk of chronic diseases like chronic kidney disease and chronic obstructive pulmonary disease (COPD) (3).

The yearly global incidence of sepsis is approximately 30 million, resulting in 6 million deaths (4). However, obesity unexpectedly appears to protect against death from all causes in individuals with sepsis (5). This was called “reverse epidemiology” or the “obesity paradox” (6, 7). Previous studies found that overweight and obese individuals had much lower mortality rates associated with sepsis than normal-weight individuals (5, 8, 9). Research has provided insights into the link between body mass index (BMI), sepsis-associated death, and age (10); however, a specific gender-based impact of BMI on sepsis-related mortality and the gender-specific link between BMI and sepsis-associated mortality in individuals has not been explicitly addressed.

A critical analysis of existing research reveals methodological and sample characteristic differences, which brings the reliability of these studies to question. To address this knowledge gap, the current study explored the relationship between BMI, gender, and sepsis mortality, overcoming previously identified limitations. This offers a new insight into the intricate interplay of obesity, gender, and sepsis mortality.

In addition, previous research frequently regards BMI as either a continuous or categorical factor, which fails to fully capture the intricate dose–response correlation between BMI and sepsis-associated mortality. The present research work deviated from traditional methods by utilizing restricted cubic splines (RCSs) to elucidate the dose–response relationship. After comprehensively evaluating for the obesity paradox in a cohort of patients with sepsis, the main objective was to get a detailed comprehension of a possible obesity paradox in sepsis prognosis. Investigate for differences in the association between BMI and mortality in men versus women.

## Methods

### Study design

The current work adhered to the standards outlined in the STROBE statement. This retrospective investigation of patients with sepsis was longitudinal and single-center.

### Patient and public involvement

The study did not include active participation from patients or the general public.

### Data source

The Medical Information Mart for Intensive Care IV (MIMIC-IV V2.2) database is a carefully curated and identifiable collection of medical records from patients hospitalized in the intensive care unit (ICU) between 2008 and 2019. The authorization to utilize MIMIC-IV data for research (Certification Number: 38807989) was secured, having satisfactorily concluded the National Institutes of Health Protecting Human Research Participants training course. The Ethics Committee of Kunshan First People’s Hospital approved the study (Ethics Number 2023–04-001-K02).

### Study population

The study comprised 14,883 individuals diagnosed with sepsis who had BMI information. Participants were recruited from the MIMIC-IV database (11). The Third International Consensus Definitions for Sepsis and Septic Shock (Sepsis-3) guidelines were used to describe sepsis. The initial screening included patients with a sepsis diagnosis, including those with sepsis, severe sepsis, and septic shock (ICD9 codes: 99591, 99,592, 78,552, respectively). Exclusion criteria included patients who were under 18 years old and had ICU stays of less than 24 h. For multiple admissions, only the first ICU admission records were considered.

### Data retrieval

The extraction of data was executed with Structured Query Language (SQL). Patients’ age, sex, race, BMI, and Charlson comorbidity index were recorded. Records about the administration of vasopressors, mechanical ventilation, and sedatives within the first 24 h after admission to the ICU were also collected. Comorbidity data, such as diabetes mellitus, congestive heart failure (CHF), coronary artery disease (CAD), hypertension, stroke, renal disease, atrial fibrillation (AFIB), liver disease, chronic pulmonary disease, and malignant tumor, were gathered using the International Classification of Diseases coding systems. The initial data collected at the onset of sepsis included vital signs (heart rate and minimum arterial pressure), the severity of illness [Simplified Acute Physiologic Score (SAPS), laboratory tests partial pressure of oxygen (PO_2_), hemoglobin concentration, white blood cell count, lactate, creatinine, glucose and pH levels] and sequential organ failure assessment (SOFA), were retrieved.

### Exposure and outcomes

The exposure was BMI, calculated as weight (kg)/height^2^ (m^2^). The primary outcome assessed was the mortality due to any cause within 28 days. Secondary outcomes examined were the mortality after 1 year and the duration of stay in the ICU.

### Statistical analysis

This retrospective study did not use *a priori* statistical analysis strategy and statistical power calculation. Based on the data that was already present in the database, the sample size was selected. The primary indicator for the research was BMI. All missing values, entries with recording errors, and other potential confounding factors with missing values exceeding 10% were excluded.

Supplementary Table S1 shows the variable missing rates. Missing values for each variable were estimated using multiple imputations (9). Multicollinearity among variables was detected using the variance inflation factor. The absence of multicollinearity for each variable was indicated by a variance inflation factor of <5 (Supplementary Table S2).

BMI was categorized into underweight (less than 18.5 kg/m^2^), normal weight (18.5–24.9 kg/m^2^), overweight (25–29.9 kg/m^2^), and obese (more than 30 kg/m^2^) using the World Health Organization (WHO) standards. Fifteen patients were excluded due to presumed erroneous data defined as BMI > 100 kg/m^2^. The patients were also grouped according to age as <60, 60–80, and ⩾ 80. Categorical variables were represented using numbers and percentages, and between-group differences were found using chi-squared and Fisher’s exact tests. Continuous variables were expressed using medians and interquartile-range values, and between-group differences were identified using the Mann–Whitney U test. Multivariable logistic regression models were used to evaluate the association between different BMI categories and 28-day mortality in sepsis patients. We constructed two regression models to control for confounding biases by adjusting for covariates. The selection of covariates was driven both theoretically and statistically. Some covariates, theoretically associated with mortality, were fixed in the model, such as age, gender, race, SAPS, SOFA, and Charlson Comorbidity Index. Other variables were selected using statistical methods. First, variables with variance inflation factors greater than 5 were excluded to avoid multicollinearity. We constructed both unadjusted and adjusted models. The adjusted model included age, gender, race, SAPS, SOFA, Charlson comorbidity index, diabetes, hypertension, coronary artery disease, congestive heart failure, atrial fibrillation, malignancy, stroke, chronic obstructive pulmonary disease, renal disease, liver disease and glucose. The nonlinear relationship between BMI and 28-day all-cause mortality was assessed using RCSs Nonlinear model knots were used to distribute BMI into quartiles. The nonlinear association between BMI and patient all-cause mortality was analyzed, and its *p-*value was calculated.

A subgroup analysis of sex and age was also performed to explore potential relationships within specific subgroups.

The statistical analyses were conducted using R software (version 4.2.3; R Foundation for Statistical Computing, Vienna, Austria) and Empowerstats.^1^ Statistical significance was determined at a two-tailed *p*-value of less than 0.05.

## Results

### Patient selection

Supplementary Figure S1 outlines the patient selection process identifying 25,599 records. The cohort consisted of 14,883 patients after excluding unqualified records.

### Demographic and hospitalization characteristics by BMI

Table 1 summarizes the demographic and hospitalization characteristics of male patients with sepsis (*n* = 9,022). A significant age difference exists among the BMI categories (*p* < 0.001). Older age showed a significant association with lower BMI and vice versa. Remarkably, BMI significantly impacts disease severity, as evidenced by SAPS (*p* < 0.001) and the Charlson comorbidity index (*p* < 0.001). This demonstrates an inverse relationship between lower BMI and higher scores on SAPS and the Charlson comorbidity index. Significant differences in comorbidities (*p* < 0.001) were observed, including atrial fibrillation, diabetes, renal disease, COPD, hypertension, liver disease, and metastatic cancer. Key vital signs and laboratory parameters exhibited significant differences among BMI categories. Clinical interventions, such as mechanical ventilation and vasopressor use, displayed BMI-related variations (*p* < 0.001).

**Table 1:** Demographic information and hospitalization characteristics of male sepsis patients.

Demographic or hospitalization characteristic	Overall *n* = 9,022	Healthy weight (18.5–24.9 kg/m^2^); *n* = 2,441	Underweight (<18.5 kg/m^2^); *n* = 186	Overweight (25.0–29.9 kg/m^2^); *n* = 3,276	Obese (≥30.0 kg/m^2^); *n* = 3,119	*P-*value
Age (years)	63.6 ± 15.1	65.3 ± 16.8	65.4 ± 17.1	64.7 ± 15.0	61.1 ± 13.4	< 0.001
Age, n (%)						< 0.001
<60	3,207 (35.5)	791 (32.4)	60 (32.3)	1,084 (33.1)	1,272 (40.8)	
60–80	4,442 (49.2)	1,093 (44.8)	80 (43)	1,638 (50)	1,631 (52.3)	
≥80	1,373 (15.2)	557 (22.8)	46 (24.7)	554 (16.9)	216 (6.9)	
Race and ethnicity, n (%)						< 0.001
Asian	239 (2.7)	130 (5.3)	4 (2.2)	76 (2.3)	29 (0.9)	
Black	563 (6.2)	188 (7.7)	28 (15.1)	159 (4.9)	188 (6)	
Hispanic	307 (3.4)	98 (4)	8 (4.3)	105 (3.2)	96 (3.1)	
White	6,191 (68.7)	1,569 (64.3)	109 (58.6)	2,320 (70.9)	2,193 (70.3)	
Unknown/Other	1716 (19.0)	454 (18.6)	37 (19.9)	612 (18.7)	613 (19.7)	
SAPS score	37.0 (29.0, 47.0)	37.0 (30.0, 47.0)	40.0 (32.2, 48.0)	36.0 (29.0, 46.0)	37.0 (29.0, 47.0)	< 0.001
SOFA	2.0 (0.0, 4.0)	2.0 (0.0, 4.0)	1.0 (0.0, 3.0)	2.0 (0.0, 4.0)	2.0 (0.0, 4.0)	0.01
Charlson comorbidity index	5.0 (3.0, 7.0)	5.0 (3.0, 7.0)	6.0 (4.0, 8.0)	4.0 (3.0, 7.0)	4.0 (3.0, 6.0)	< 0.001
Comorbidity						
AFIB, n (%)	3,110 (34.5)	818 (33.5)	46 (24.7)	1,161 (35.4)	1,085 (34.8)	0.016
Diabetes, n (%)	2,847 (31.6)	565 (23.1)	46 (24.7)	922 (28.1)	1,314 (42.1)	< 0.001
CHF, n (%)	1,461 (16.2)	390 (16)	29 (15.6)	513 (15.7)	529 (17)	0.537
Renal disease, n (%)	7,445 (82.5)	1864 (76.4)	143 (76.9)	2,656 (81.1)	2,782 (89.2)	< 0.001
COPD, n (%)						< 0.001
	543 (6.0)	146 (6)	23 (12.4)	165 (5)	209 (6.7)	
Hypertension, n (%)	4,075 (45.2)	933 (38.2)	55 (29.6)	1,550 (47.3)	1,537 (49.3)	< 0.001
CAD, n (%)	1,186 (13.1)	304 (12.5)	20 (10.8)	416 (12.7)	446 (14.3)	0.104
Stroke, n (%)	874 (9.7)	252 (10.3)	16 (8.6)	325 (9.9)	281 (9)	0.359
Liver disease, n (%)	2,123 (23.5)	581 (23.8)	64 (34.4)	706 (21.6)	772 (24.8)	< 0.001
Metastatic cancer, n (%)	2036 (22.6)	611 (25)	62 (33.3)	794 (24.2)	569 (18.2)	< 0.001
Vital signs						
MAP (mm Hg)	53.7 ± 11.3	53.0 ± 11.5	51.6 ± 12.5	54.4 ± 10.9	53.8 ± 11.5	< 0.001
Heart rate (beats/min)	116.3 ± 24.4	116.7 ± 23.8	121.6 ± 30.8	115.0 ± 24.5	117.1 ± 24.3	< 0.001
Laboratory tests						
PO2 (mmHg)	67.0 (42.0, 95.0)	67.0 (41.0, 104.0)	48.0 (36.0, 90.8)	71.0 (44.0, 99.0)	64.0 (42.0, 87.0)	< 0.001
Lactate (mmol/L)	2.4 (1.7, 3.7)	2.5 (1.7, 3.8)	2.1 (1.6, 3.4)	2.4 (1.8, 3.6)	2.4 (1.7, 3.7)	0.08
Hemoglobin (g/dL)	8.7 ± 1.9	8.5 ± 1.8	8.2 ± 1.8	8.7 ± 1.9	8.9 ± 2.0	< 0.001
pH	7.3 ± 0.1	7.3 ± 0.1	7.3 ± 0.1	7.3 ± 0.1	7.3 ± 0.1	< 0.001
Creatinine (mg/dL)	1.1 (0.9, 1.7)	1.0 (0.8, 1.6)	1.0 (0.8, 2.0)	1.1 (0.9, 1.6)	1.2 (0.9, 1.9)	< 0.001
White blood cell counts (×10^9^/L)	16.0 (12.0, 21.2)	15.5 (11.6, 20.6)	16.0 (11.5, 21.5)	15.7 (11.9, 20.5)	16.7 (12.5, 21.9)	< 0.001
Glucose, (mg/dL)	131.0 (108.0, 166.0)	124.0 (103.0, 156.0)	123.0 (103.0, 152.8)	131.0 (108.0, 164.0)	138.0 (112.0, 177.0)	< 0.001
Interventions						
Mechanical ventilation use, n (%)	8,459 (93.8)	2,233 (91.5)	170 (91.4)	3,089 (94.3)	2,967 (95.1)	< 0.001
Vasopressor use, n (%)	5,552 (61.5)	1,464 (60)	100 (53.8)	2038 (62.2)	1950 (62.5)	0.027
Sedative use, n (%)	635 (7.0)	156 (6.4)	8 (4.3)	225 (6.9)	246 (7.9)	0.064

Continuous Variables: Normally distributed variables are shown as mean (± SD), and non-normally distributed variables as median (IQR). Categorical Variables: Categorical variables are presented as counts and percentages (%). BMI, Body Mass Index; MAP, Mean Arterial Pressure; SAPS, Simplified Acute Physiology Score; SOFA, Sequential Organ Failure Assessment; COPD, Chronic obstructive pulmonary disease; CAD, Coronary artery disease; CHF, Congestive heart failure; AFIB, Atrial fibrillation.

Table 2 presents the demographic and hospitalization characteristics of female patients with sepsis (*n* = 5,861). Similar to males, older age was significantly associated with lower BMI (*p* < 0.001). Both SOFA scores (*p* = 0.047) and the Charlson comorbidity index (*p* = 0.044) showed significant differences across BMI groups. Although the median Charlson comorbidity index was consistent at 5.0 across BMI groups, further analysis suggested that females with lower BMI tended to have a higher burden of comorbidities, which is reflected in the significant differences observed across the BMI categories. Significant differences in comorbidities (*p* < 0.001) were observed, including diabetes, renal disease, COPD, and hypertension. BMI-related differences were noted in the vital signs and laboratory parameters. Clinical interventions, such as mechanical ventilation, exhibited BMI-related variations (*p* < 0.001).

**Table 2:** Demographic information and hospitalization characteristics of female sepsis patients.

Demographic or hospitalization characteristic	Overall *n* = 5,861	Healthy weight (18.5–24.9 kg/m^2^); *n* = 1,767	Underweight (<18.5 kg/m^2^); *n* = 245	Overweight (25.0–29.9 kg/m^2^); *n* = 1,632	Obese (≥30.0 kg/m^2^); *n* = 2,217	*P-*value
Age (years)	66.2 ± 16.0	67.6 ± 17.1	68.9 ± 16.8	67.3 ± 16.5	64.1 ± 14.4	< 0.001
Age, n (%)						< 0.001
<60	1775 (30.3)	511 (28.9)	57 (23.3)	470 (28.8)	737 (33.2)	
60–80	2,771 (47.3)	751 (42.5)	113 (46.1)	726 (44.5)	1,181 (53.3)	
≥80	1,315 (22.4)	505 (28.6)	75 (30.6)	436 (26.7)	299 (13.5)	
Race and ethnicity, n (%)						< 0.001
Asian	124 (2.1)	68 (3.9)	7 (2.9)	35 (2.1)	14 (0.6)	
Black	568 (9.7)	143 (8.1)	25 (10.2)	150 (9.2)	250 (11.3)	
Hispanic	173 (3.0)	33 (1.9)	1 (0.4)	54 (3.3)	85 (3.8)	
White	3,992 (68.2)	1,208 (68.4)	171 (69.8)	1,127 (69.2)	1,486 (67.1)	
Unknown/Other	997 (17.0)	313 (17.7)	41 (16.7)	263 (16.1)	380 (17.2)	
SAPS score	38.0 (30.0, 48.0)	38.0 (30.0, 48.0)	40.0 (31.0, 52.0)	38.0 (30.0, 48.0)	38.0 (30.0, 48.0)	0.135
SOFA	1.0 (0.0, 3.0)	1.0 (0.0, 3.0)	1.0 (0.0, 3.0)	1.0 (0.0, 3.0)	1.0 (0.0, 4.0)	0.047
Charlson comorbidity index	5.0 (3.0, 7.0)	5.0 (3.0, 7.0)	5.0 (4.0, 7.0)	5.0 (3.0, 7.0)	5.0 (3.0, 7.0)	0.044
Comorbidity						
AFIB, n (%)	1890 (32.2)	588 (33.3)	69 (28.2)	530 (32.5)	703 (31.7)	0.382
Diabetes, n (%)	1739 (29.7)	341 (19.3)	32 (13.1)	443 (27.1)	923 (41.6)	< 0.001
CHF, n (%)	1,085 (18.5)	331 (18.7)	36 (14.7)	321 (19.7)	397 (17.9)	0.22
Renal disease, n (%)	4,748 (81.0)	1,294 (73.2)	171 (69.8)	1,293 (79.2)	1990 (89.8)	< 0.001
COPD, n (%)	491 (8.4)	137 (7.8)	30 (12.2)	118 (7.2)	206 (9.3)	0.012
Hypertension, n (%)	2,672 (45.6)	728 (41.2)	90 (36.7)	761 (46.6)	1,093 (49.3)	< 0.001
CAD, n (%)	482 (8.2)	132 (7.5)	16 (6.5)	134 (8.2)	200 (9)	0.248
Stroke, n (%)	668 (11.4)	224 (12.7)	26 (10.6)	193 (11.8)	225 (10.1)	0.081
Liver disease, n (%)	1,302 (22.2)	370 (20.9)	62 (25.3)	364 (22.3)	506 (22.8)	0.321
Metastatic cancer, n (%)	1,382 (23.6)	475 (26.9)	76 (31)	357 (21.9)	474 (21.4)	< 0.001
Vital signs						
MAP (mm Hg)	51.3 ± 11.0	51.7 ± 11.3	51.2 ± 11.7	51.3 ± 10.9	50.9 ± 10.9	0.132
Heart rate (beats/min)	118.7 ± 24.4	119.6 ± 24.6	120.5 ± 24.4	117.5 ± 24.3	118.6 ± 24.2	0.044
Laboratory tests						
PO2 (mmHg)	62.0 (39.0, 90.0)	63.0 (40.0, 94.0)	51.5 (36.8, 86.5)	64.0 (39.0, 92.0)	61.0 (39.0, 85.0)	0.003
Lactate (mmol/L)	2.5 (1.6, 3.9)	2.4 (1.6, 3.9)	2.1 (1.5, 3.5)	2.5 (1.6, 3.9)	2.6 (1.6, 4.0)	0.045
Hemoglobin (g/dL)	8.1 ± 1.7	8.0 ± 1.7	8.2 ± 1.7	8.1 ± 1.7	8.2 ± 1.8	0.145
pH	7.3 ± 0.1	7.3 ± 0.1	7.3 ± 0.1	7.3 ± 0.1	7.3 ± 0.1	< 0.001
Creatinine (mg/dL)	0.9 (0.7, 1.5)	0.8 (0.6, 1.3)	0.9 (0.6, 1.4)	0.9 (0.7, 1.4)	1.0 (0.7, 1.6)	< 0.001
White blood cell counts (×10^9^/L)	16.2 (12.1, 21.7)	16.0 (11.9, 21.7)	15.2 (11.1, 20.2)	15.8 (11.8, 21.3)	16.7 (12.6, 22.1)	<0.001
Glucose (mg/dL)	133.0 (108.0, 170.0)	126.0 (103.0, 157.0)	127.0 (104.0, 160.0)	131.0 (107.0, 166.0)	141.0 (113.0, 181.0)	< 0.001
Interventions						
Mechanical ventilation use, n (%)	5,390 (92.0)	1,583 (89.6)	222 (90.6)	1,499 (91.9)	2086 (94.1)	< 0.001
Vasopressor use, n (%)	3,354 (57.2)	977 (55.3)	128 (52.2)	952 (58.3)	1,297 (58.5)	0.058
Sedative use, n (%)	422 (7.2)	118 (6.7)	18 (7.3)	115 (7)	171 (7.7)	0.646

Continuous Variables: Normally distributed variables are shown as mean (± SD), and non-normally distributed variables as median (IQR). Categorical Variables: Categorical variables are presented as counts and percentages (%). BMI, Body Mass Index; MAP, Mean Arterial Pressure; SAPS, Simplified Acute Physiology Score; SOFA, Sequential Organ Failure Assessment; COPD, Chronic obstructive pulmonary disease; CAD, Coronary artery disease; CHF, Congestive heart failure; AFIB, Atrial fibrillation.

### Comparison of outcomes in different genders

Table 3 and Supplementary Table S3 presents the patient outcomes stratified by BMI and age categories. There were significant differences in the duration of ICU stay and the rates of death at 28 days and 1 year among male patients, depending on their BMI categories (*p* < 0.001), with obese patients having the longest mean ICU stay of 3.4 (1.7, 8.0) days. Underweight male patients experienced the highest 28-day (22%) and 1-year (53.2%) mortality. Obese males had the lowest risk of death, with a 28-day and 1-year mortality of 12.5 and 22.3%, respectively.

**Table 3:** Mortality in different BMI patients with sepsis.

Outcome	Overall	Healthy weight (18.5–24.9 kg/m^2^)	Underweight (<18.5 kg/m^2^)	Overweight (25.0–29.9 kg/m^2^)	Obese (≥30.0 kg/m^2^)	*P-*value
Male	*n* = 9,022	*n* = 2,441	*n* = 186	*n* = 3,276	*n* = 3,119	
Time in ICU (days)	3.2 (1.5,7.1)	3.3 (1.7, 7.0)	3.3 (2.0, 7.7)	3.1 (1.4, 6.5)	3.4 (1.7, 8.0)	< 0.001
28-day mortality, n (%)	1,279 (14.2)	436 (17.9)	41 (22)	413 (12.6)	389 (12.5)	< 0.001
1-year mortality, n (%)	2,403 (26.6)	838 (34.3)	99 (53.2)	770 (23.5)	696 (22.3)	< 0.001
Female	*n* = 5,861	*n* = 1767	*n* = 245	*n* = 1,632	*n* = 2,217	
Time in ICU (days)	3.7 (1.9,7.8)	3.7 (1.9, 7.8)	3.9 (1.9, 7.3)	3.6 (1.7, 7.6)	3.8 (2.0, 8.1)	0.108
28-day mortality, n (%)	1,000 (17.1)	304 (17.2)	69 (28.2)	279 (17.1)	348 (15.7)	< 0.001
1-year mortality, n (%)	1,842 (31.4)	609 (34.5)	108 (44.1)	490 (30)	635 (28.6)	< 0.001

In female patients, ICU stay did not differ between BMI groups (*p* = 0.108). However, 28-day and 1-year mortality varied substantially by BMI (*p* < 0.001). Underweight females had markedly elevated mortality, with 28-day and 1-year rates of 28.2 and 44.1%, respectively. Obese females experienced relatively lower mortality of 15.7% at 28 days and 28.6% at 1 year.

### Gender differences in the correlation between BMI and mortality

Supplementary Table S4 presents the multivariable logistic regression analysis results showing the relationship between mortality and BMI. After accounting for potential confounders that might influence the results (Supplementary Table S5), each unit rise in BMI was linked to 2% lower odds of 28-day mortality [adjusted odds ratio (OR) = 0.98, 95% confidence interval (CI) = 0.98, 0.99, *p* < 0.001]. When stratified by gender, the outcomes showed a significant correlation (*p* for interaction = 0.014). For females, being underweight was linked to 80% higher odds of 28-day mortality in comparison to having a normal weight (adjusted OR = 1.80, 95% CI = 1.28, 2.53, *p* = 0.001). No significant relationships were seen in the overweight and obese categories. In males, both overweight and obesity were linked to 33% lower odds of 28-day mortality relative to normal weight. The adjusted OR were 0.67 (95% CI = 0.57, 0.79, *p* < 0.001) for overweight and 0.67 (95% CI = 0.57, 0.80, *p* < 0.001) for obesity (Supplementary Table S6).

### RCS analyses of nonlinear relationships

**Figure 1:**
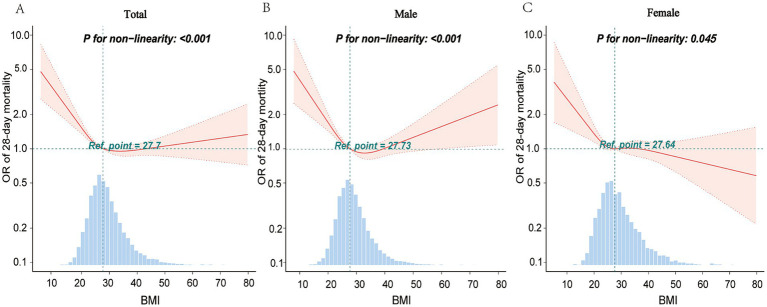
Restricted spline curves for the association between BMI and 28-day mortality. The graphs present odds ratios (ORs) for 28-day mortality, adjusting for age, gender, race, SAPS, SOFA, and the Charlson comorbidity index. It also adjusts for various comorbidities, including hypertension, stroke, congestive heart failure, renal disease, diabetes, chronic obstructive pulmonary disease, atrial fibrillation, malignancy cancer, coronary artery disease, and liver disease. Additionally, the analysis was adjusted for blood glucose levels to account for its potential impact on the outcomes. Logistic regression modeling facilitated data fitting. Solid lines on the graphs denote ORs, with shaded areas representing 95% confidence intervals (CIs). OR, odds ratio; CI, confidence interval.

RCS models were developed to evaluate the link between BMI and mortality. Figure 1 depicts the dose–response curves demonstrating the correlation between BMI and 28-day all-cause mortality. The curves were obtained after performing logistic analysis and adjusting for significant covariates. The dose–response analysis demonstrated a L-shaped curve depicting the relationship between BMI and the risk of 28-day all-cause mortality (*p* for nonlinearity <0.001) (Figure 1A).

According to subgroup analysis, there was significant variation in the link between BMI and 28-day mortality in males versus females. RCS models were constructed for sex-stratified analysis. Figure 1B shows a U-shaped curve relationship between BMI and 28-day all-cause mortality in male patients with sepsis (*p* for nonlinearity <0.001). However, it reveals an L-shaped correlation between these two factors in the female patients (*p* for nonlinearity = 0.045) (Figure 1C). In our cohort, there were 3,418 patients with late-stage cancer. The results of a secondary analysis excluding these patients were consistent with the original findings, indicating that the inclusion of late-stage cancer patients did not significantly alter the overall results. Analyses were also conducted of the results in different age groups and Charlson comorbidity index score groups (Supplementary Figures S2, S3). This study produced curve-fitting graphs for BMI and 28-day mortality, considering various factors such as gender, age, and Charlson comorbidity index scores (Supplementary Figure S4).

### Change points and associations in the BMI-mortality relationship

Table 4 presents the estimated change points associated with BMI and 28-day mortality. For the entire cohort, a BMI change point was identified at 27.89 kg/m^2^. Below this change point, each 1 kg/m^2^ increment in the BMI was linked with an OR of 0.94 (95% CI = 0.92, 0.96, *p* < 0.001) for mortality, indicating a protective effect. Above this BMI threshold, the OR per 1 kg/m^2^ increase was neutral at 1.01 (95% CI = 1.00, 1.02, *p* = 0.283), suggesting no additional risk. Gender-specific analyses revealed a higher BMI change point for males (29.22 kg/m^2^) than females (20.67 kg/m^2^). The protective association below the change point was more pronounced in the males (OR = 0.93, 95% CI = 0.91, 0.95, *p* < 0.001) than in the overall cohort, with a slight increase in mortality risk above the change point (OR = 1.02, 95% CI = 1.00, 1.03, *p* = 0.030). Females exhibited a more substantial protective effect below their lower change point (OR = 0.86, 95% CI = 0.80, 0.93, *p* < 0.001), with a neutral effect above it (OR = 0.99, 95% CI = 0.98, 1.00, *p* = 0.129). Stratifying by age and Charlson comorbidity index revealed variability in change points and effect sizes but consistently showed a relation between lower BMI and higher mortality risk.

**Table 4:** Estimated change points from piecewise two-line models in the relationship between BMI and mortality and the associations with mortality below and above the change point.

	BMI change point, kg/m^2^ (95% CI)	OR per 1 kg/m^2^ BMI increase below change point (95% CI)	*P-*value	OR per 1 kg/m^2^ BMI increase above change point (95% CI)	*P-*value
All sepsis patients	27.89	0.94 (0.92, 0.96)	<0.001	1.01(1.00, 1.02)	0.283
Male (*n* = 9,022)	29.22	0.93 (0.91, 0.95)	<0.001	1.02 (1.00, 1.03)	0.030
Age					
<60 (3,207)	29.14	0.95 (0.91, 0.99)	0.014	1.02 (1.00, 1.04)	0.070
60–80 (4,442)	26.68	0.90 (0.87, 0.94)	<0.001	1.00 (0.98, 1.02)	0.936
⩾80 (1,373)	29.22	0.9 (0.88, 0.96)	<0.001	1.03 (0.96, 1.10)	0.389
Charlson comorbidity index					
<6	27.85	0.92 (0.88, 0.95)	<0.001	1.02 (1.00, 1.04)	0.063
⩾6	29.80	0.93 (0.91, 0.96)	<0.001	1.01 (0.99, 1.03)	0.317
Female (*n* = 5,861)	20.67	0.86 (0.80, 0.93)	<0.001	0.99 (0.98, 1.00)	0.129
Age					
<60 (1,775)	NA	1.00 (0.98, 1.02)	0.749		
60–80 (2,771)	20.76	0.85 (0.77, 0.95)	0.004	0.99 (0.97, 1.00)	0.127
⩾80 (1,315)	20.53	0.74 (0.63, 0.86)	<0.001	0.99 (0.97, 1.02)	0.696
Charlson comorbidity index					
<6	22.31	0.82 (0.76, 0.89)	<0.001	0.99 (0.97, 1.01)	0.276
⩾6	NA	0.99 (0.98, 1.01)	0.458		

OR, odds ratio; NA, not available.

## Discussion

Our study data indicate a correlation between BMI and mortality in sepsis patients. The findings suggest that low BMI may have adverse consequences, as underweight individuals exhibited higher mortality rates. Conversely, obese patients showed lower mortality rates, suggesting a potential protective effect of higher BMI. The relationship between 28-day mortality and BMI demonstrated an L-shaped curve, with significant gender-specific differences. Specifically, females exhibited an L-shaped relationship, while males exhibited a U-shaped relationship. The BMI-mortality relationship in sepsis patients was analyzed using piecewise two-line models to estimate change points and mortality associations on either side of these points. The results emphasize an intricate relationship that relies on BMI thresholds, which differ according to gender, age, and comorbidity burden, as assessed by the Charlson comorbidity index. These results emphasize the need to consider individual patient characteristics in the BMI-mortality assessment for sepsis patients. The identified BMI change points and their differential effects reinforce the concept of an “obesity paradox,” suggesting a survival benefit for higher BMI up to a certain point.

The present study underscores that the correlation between BMI and 28-day mortality varies markedly between sexes—higher risk in underweight females and a protective effect in overweight and obese males. Consistent with another study, which showed that male participants with higher BMI exhibited a lower risk of mortality than female participants (12). In sepsis patients, a significant sex and BMI relationship was found in the current investigation. RCS analysis indicated a pronounced nonlinear relationship in males, characterized by a U-shaped curve. On the other hand, females exhibited an L-shaped curve, requiring a deeper understanding of the physiological mechanisms involved. The U-shaped curve in males may relate to the combined effects of visceral fat and inflammatory responses, potentially leading to increased mortality. In contrast, the L-shaped curve in females might reflect the protective role of subcutaneous fat on cardiovascular health and immunity. Hormones like estrogen and testosterone, which differ between genders, could influence these patterns. Specifically, estrogen’s anti-inflammatory properties might contribute to better outcomes in females (13). The present results align with growing evidence of gender-specific health responses (14, 15), highlighting the distinct impacts of different fat types on metabolic health in males and females. One study indicated that gender differences significantly impact the critical points of BMI based on body fat percentage (16). This suggests that gender differences should be considered in weight and BMI studies.

The relationship between BMI and mortality associated with sepsis in patients has been the subject of several investigations. An inverse relationship between BMI and sepsis-related mortality was shown in a meta-analysis of observational data (17), and a higher BMI has been associated with improved survival rates among older individuals with sepsis (12) but not younger individuals with sepsis (18). An analysis of a group of Japanese patients with severe sepsis revealed a higher rate of death within 28 days among individuals with lower BMI (< 18.5 kg/m^2^) (19). Another meta-analysis reported decreased mortality in obese and overweight patients and increased mortality in patients who were underweight (20). Retrospective cohort research involving 55,038 adult patients diagnosed with sepsis revealed a correlation between obesity (BMI ≥ 30 kg/m^2^) and survival rates in sepsis, resulting in an absolute mortality reduction (21). Mica et al. proposed that fatty tissue associated with higher BMI exerts a protective effect against inflammatory reactions like sepsis, and elevations in leptin and inflammatory biomarkers like C-reactive protein within the obese population have been considered as having a mechanistic role in the obesity paradox (22–24).

However, the inverse relationship between BMI and sepsis-related mortality has been challenged by some studies indicating the opposite. In a retrospective cohort research involving 834 individuals with sepsis admitted to the ICU, obese patients had higher mortality rates than non-obese patients (23). Impaired immune function in the obese population causes a significant increase in mortality rates (25). Population-based cohort research conducted on 0.5 million Chinese adults found that compared with a reference BMI of 22.5 to <25.0 kg/m^2^, the multivariable-adjusted hazard ratios for sepsis-related mortality were 2.42 for BMI < 18.5, 1.59 for 18.5 to <20.0, 1.21 for 20.0 to <22.5, 0.97 for 25.0 to <27.5, 0.98 for 27.5 to <30.0, and 1.22 for ≥30.0 kg/m^2^. The study also found increased sepsis-related mortality risk even in participants with low-and mid-normal weight (26). A two-sample Mendelian randomization study found that sepsis mortality at 28 days increased with increasing BMI; however, the effect disappeared at 90 days (27, 28). Confounders in causal inference in observational studies can lead to opposite conclusions. The correlation between BMI and mortality in sepsis patients is intricate, and the results from different research are inconclusive. Hence, the influence of BMI on sepsis-associated mortality may be contingent upon several elements and necessitates more investigation to achieve a thorough comprehension.

Although our data suggest that the overweight and obese groups had better survival chances compared to those with lower BMI, caution is warranted in interpreting these findings. Our analysis revealed that lower BMI groups had higher comorbidity scores, indicating that individuals with lower BMI may have more severe underlying health conditions, such as cachexia, which could negatively impact their prognosis. Therefore, the protective effect of a higher BMI may be partially attributable to the relatively better health status of these individuals. Although high BMI can predispose individuals to severe conditions, our findings indicate that individuals with higher BMI generally have better outcomes compared to those with severe comorbidities and lower BMI. This aligns with studies on acute respiratory distress syndrome (ARDS), where higher BMI was linked to better outcomes. Other pathophysiological mechanisms described in recent literature (29) may also explain these associations.

The study partially supports the existence of an obesity paradox in sepsis patients (30). Employing RCS, more precise ORs were derived. A lower OR was identified for BMI values of 27.7–42 kg/m^2^, which correlates with an increased survival rate. Multifactorial analysis revealed a lower mortality rate in the obese population. In contrast, curve-fitting results indicated that males with higher BMI had a higher mortality rate. The lower mortality rate in the obese population may be attributed to the higher prevalence of mild obesity. However, severe obesity was linked to a higher mortality rate. The curve-fitting results suggest that a more significant proportion of people with moderate obesity was responsible for the overall decrease in the death rate.

A significant association was found between elevated BMI and decreased death rates in comparison to the healthy BMI range recommended by the WHO, and given the age of the present study population was 64.66 ± 15.55 years, this fits with previous study results (12, 18). Aging leads to a redistribution of body composition, including a decrease in lean body mass and bone density, particularly with an increase in abdominal fat mass. A study revealed that the association between BMI and mortality is influenced, to some extent, by the associations of lean body mass and fat mass with mortality (31). Body composition markers, such as waist circumference, might reveal varying impacts on specific and overall mortality causes, offering a new understanding of the negative relationship between BMI and mortality (31–33). Our dataset lacked certain reliable indicators of mortality such as lean body mass and central obesity, and future studies on mortality and sepsis should include such markers of body composition. However, analyses from a study also indicated that a higher BMI correlates with lower mortality rates in older individuals, suggesting an increased demand for nutritional reserves in older age (34). These findings underscore the consideration of age in healthy weight recommendations, emphasizing the need for further research to determine the benefits of weight gain in the elderly.

### Limitations

The present study provides insights into the link between BMI and gender-specific 28-day sepsis-associated mortality in patients. However, the current research has several limitations. First, as a retrospective analysis, the study is subject to information bias, which could result in inaccuracies or incomplete data. In particular, lipid variables such as Low-Density Lipoprotein cholesterol and triglycerides, which may be strongly associated with BMI, were excluded from the primary analysis due to a high rate of missing data. The omission of these variables may limit our ability to fully adjust for metabolic factors that influence the relationship between BMI and sepsis mortality. Second, BMI was employed to measure overall obesity instead of waist circumference, which assesses central obesity. Observational studies indicate that there is a correlation between abdominal obesity and a higher likelihood of death linked to sepsis (35). Despite using RCS to obtain more precise ORs, unmeasured confounders may still influence the BMI-mortality relationship. Moreover, this study failed to include variables such as muscle mass, fat distribution, and other aspects of body composition. These factors could impact the prognosis of sepsis patients. Other potential influencers such as lifestyle, diet, socioeconomic status, and baseline health conditions were also not examined, which could also impact the observed BMI-mortality relationship. The identification of sepsis-3 patients was primarily based on ICD-9 codes, which, although operationally convenient and readily available, may not be as precise as incorporating the SOFA score. The use of ICD-9 codes could potentially include patients with milder symptoms, which might influence the assessment of the severity of sepsis. However, to enhance the accuracy of our study’s findings, our primary analysis outcomes were adjusted for the SOFA score to control for potential biases. Finally, as an observational study, causality cannot be inferred. The mechanism of the obesity paradox in sepsis is still being studied and could include inflammation (36–38), which was not controlled for in this study using inflammatory biomarkers. Despite these limitations, the study offers valuable insights into the complex link between BMI and mortality in sepsis patients. Future research should overcome these limitations with prospective designs, broader populations, and more comprehensive health metrics to further explore the role of BMI in the clinical management of sepsis.

### Clinical considerations and implications

The discovery of gender-specific patterns has significant clinical implications. It underscores the importance of personalized treatment to account for gender-related physiological differences in sepsis management. Future studies should investigate the roles of inflammatory biomarkers, hormones and fat distribution in sepsis outcomes to refine gender-specific treatment approaches. The current study highlights the importance of considering gender-specific BMI and mortality relationships, reinforcing the need for personalized interventions in the management of sepsis.

## Conclusion

This study uncovers the correlation between BMI and gender-specific mortality in a cohort of patients with sepsis in whom the presence of the obesity paradox was confirmed. The RCS analysis revealed a distinct L-shaped correlation between BMI and 28-day mortality. Regarding sex stratification, a U-shaped correlation was observed between BMI and 28-day mortality in male patients, whereas an L-shaped correlation was seen in females. It is necessary to conduct further studies, including many centers and large samples, in order to examine the disparities between sexes in the obesity paradox.

## Data Availability

The raw data supporting the conclusions of this article will be made available by the authors, without undue reservation.

## Footnotes

^1^http://www.empowerstats.com
