# Supplementary material for: Correlation between body mass index and gender-specific 28-day mortality in patients with sepsis: a retrospective cohort study
Source: Front Med (Lausanne). 2024 Oct 8;11:1462637. doi: 10.3389/fmed.2024.1462637 (PMC11493596; doi:10.3389/fmed.2024.1462637)
Supplement: Supplementary file 1 [file Data_Sheet_1.docx]

**ELECTRONIC SUPPLEMENTARY MATERIAL**

**Title: Correlation between body mass index and gender-specific 28-day mortality in patients with sepsis: a retrospective cohort study**

TABLE OF CONTENTS

**Supplemental Table S1. Missing data.**

**Supplemental Table S2. Multicollinearity analysis.**

**Supplemental table S3. Mortality in Different BMI and Age Groups of Sepsis Patients**

**Supplemental table S4. Multivariable Logistic analysis.**

**Supplemental table S5. Univariate Logistic analysis.**

**Supplemental table S6. Subgroup analysis.**

**Supplemental Figure S1**. **Flowchart of patient selection.**

**Supplemental Figure S2: Analysis of the correlation between BMI and 28-day mortality.**

**Supplemental Figure S3: Analysis of the correlation between BMI and 28-day mortality.**

**Supplemental Figure S4: The smoothed curve fitting graph illustrates the relationship between BMI and the 28-day mortality rate in sepsis patients.**

**Supplement Table S1. Missing data**

| Missing variable | n | Miss.percentage% |
| --- | --- | --- |
| Hemoglobin | 34 | 0.2249 |
| Lactate | 1916 | 12.6745 |
| PO2 | 1910 | 12.6348 |
| Creatinine | 72 | 0.4763 |
| White blood cell counts | 35 | 0.2315 |
| PH | 1717 | 11.3581 |
| Heart rate | 8 | 0.0529 |
| MAP | 7 | 0.1125 |
| Glucose | 30 | 0.2016 |

Data missing values are given in Supplementary Table S1.

**Supplement Table S2. Multicollinearity analysis**

| Term1 | Coeff 1 | Change.percentage 1 | Term 2 | Coeff 2 | Change.percentage 2 | VIF | Colinearity | select | select.VIF |
| --- | --- | --- | --- | --- | --- | --- | --- | --- | --- |
| Crude | -0.02 | Ref. | Full | -0.02 | Ref. | 1.108 | 0 | Ref. | Ref. |
| Age | -0.01 | -34.5 | age | -0.02 | -1.6 | 1.795 | 0 | Yes | Yes |
| Gender | -0.02 | 0.8 | Gender | -0.01 | -4.1 | 1.032 | 0 | No | No |
| SAPS score | -0.02 | 0.9 | sapsii | -0.01 | -9.3 | 1.297 | 0 | No | No |
| SOFA | -0.02 | 7.8 | sofa | -0.02 | 1.4 | 1.21 | 0 | No | No |
| Charlson comorbidity index | -0.01 | -22.7 | charlson_comorbidity_index | -0.02 | 7 | 2.219 | 0 | Yes | Yes |
| Mechanical ventilation use | -0.02 | 8.1 | ventilation | -0.02 | -1.4 | 1.069 | 0 | No | No |
| Vasopressor use | -0.02 | 7.7 | vasopressors | -0.02 | -1.4 | 1.191 | 0 | No | No |
| Sedative use | -0.02 | -1.4 | sedative | -0.02 | 1.5 | 1.024 | 0 | No | No |
| CHF | -0.02 | 0.5 | CHF | -0.02 | 0.5 | 1.182 | 0 | No | No |
| AF | -0.02 | 0.6 | AF | -0.02 | 2.6 | 1.249 | 0 | No | No |
| Diabetes | -0.02 | -1.7 | diabetes | -0.02 | 27.7 | 1.143 | 0 | Yes | Yes |
| Hypertension | -0.02 | -8.9 | hypertension | -0.02 | 4.7 | 1.088 | 0 | No | No |
| Renal disease | -0.03 | 46.1 | renaldisease | -0.01 | -24.5 | 1.172 | 0 | Yes | Yes |
| Liver disease | -0.02 | 3.1 | liverdisease | -0.02 | 0.1 | 1.08 | 0 | No | No |
| COPD | -0.02 | 2.3 | copd | -0.02 | -2.1 | 1.168 | 0 | No | No |
| CAD | -0.02 | 0.3 | CAD | -0.02 | 0.5 | 1.082 | 0 | No | No |
| Stroke | -0.02 | -4.4 | stroke | -0.02 | 6.6 | 1.221 | 0 | No | No |
| Malignancy | -0.02 | -11 | malignancy | -0.02 | -0.1 |  |  | Yes | NA_character_ |

We observed no multicollinearity between explanatory variables.

**Supplement table S3. Mortality in Different BMI and Age Groups of Sepsis Patients**

| **Outcome** | **Overall** | **Healthy weight (18.5–24.9 kg/m²)** | **Underweight (<18.5 kg/m²)** | **Overweight (25.0–29.9 kg/m²)** | **Obese (≥30.0 kg/m²)** | *P* value |
| --- | --- | --- | --- | --- | --- | --- |
| **Male** | **n = 9022** | **n = 2441** | **n = 186** | **n = 3276** | **n = 3119** |  |
| Time in ICU (days) | 3.2 (1.5,7.1) | 3.3 (1.7, 7.0) | 3.3 (2.0, 7.7) | 3.1 (1.4, 6.5) | 3.4 (1.7, 8.0) | < 0.001 |
| Age < 60 | 3.7 (1.7, 8.5) | 3.6 (1.7, 8.1) | 3.9 (2.1, 10.3) | 3.3 (1.5, 7.5) | 3.9 (1.8, 9.3) | 0.019 |
| Age 60-80 | 3.0 (1.4, 6.6) | 3.0 (1.4, 6.9) | 3.4 (2.0, 6.2) | 2.8 (1.3, 6.1) | 3.1 (1.5, 6.9) | 0.003 |
| Age ≥80 | 3.3 (1.9, 6.2) | 3.3 (1.9, 5.8) | 3.0 (1.9, 6.2) | 3.3 (1.9, 6.1) | 3.7 (1.9, 7.2) | 0.761 |
| 28-day mortality, n (%) | 1279 (14.2) | 436 (17.9) | 41 (22) | 413 (12.6) | 389 (12.5) | < 0.001 |
| Age < 60 | 351 (10.9) | 96 (12.1) | 9 (15) | 100 (9.2) | 146 (11.5) | 0.12 |
| Age 60-80 | 594 (13.4) | 192 (17.6) | 18 (22.5) | 191 (11.7) | 193 (11.8) | < 0.001 |
| Age ≥80 | 334 (24.3) | 148 (26.6) | 14 (30.4) | 122 (22) | 50 (23.1) | 0.239 |
| 1-year mortality, n (%) | 2403 (26.6) | 838 (34.3) | 99 (53.2) | 770 (23.5) | 696 (22.3) | < 0.001 |
| Age < 60 | 651 (20.3) | 198 (25) | 25 (41.7) | 190 (17.5) | 238 (18.7) | < 0.001 |
| Age 60-80 | 1164 (26.2) | 370 (33.9) | 41 (51.2) | 369 (22.5) | 384 (23.5) | < 0.001 |
| Age ≥80 | 588 (42.8) | 270 (48.5) | 33 (71.7) | 211 (38.1) | 74 (34.3) | < 0.001 |
| **Female** | **n = 5861** | **n = 1767** | **n = 245** | **n = 1632** | **n = 2217** |  |
| Time in ICU (days) | 3.7 (1.9,7.8) | 3.7 (1.9, 7.8) | 3.9 (1.9, 7.3) | 3.6 (1.7, 7.6) | 3.8 (2.0, 8.1) | 0.108 |
| Age < 60 | 4.1 (1.9, 9.0) | 4.1 (2.0, 8.9) | 3.9 (1.7, 7.3) | 4.4 (1.9, 9.6) | 4.0 (1.9, 9.0) | 0.898 |
| Age 60-80 | 3.7 (1.8, 7.7) | 3.5 (1.8, 7.8) | 4.2 (1.9, 8.3) | 3.5 (1.5, 7.1) | 3.8 (1.9, 7.7) | 0.264 |
| Age ≥80 | 3.4 (1.9, 7.0) | 3.3 (1.8, 6.9) | 3.5 (2.0, 5.8) | 3.2 (1.7, 6.8) | 3.8 (2.2, 7.8) | 0.046 |
| 28-day mortality, n (%) | 1000 (17.1) | 304 (17.2) | 69 (28.2) | 279 (17.1) | 348 (15.7) | < 0.001 |
| Age < 60 | 218 (12.3) | 59 (11.5) | 5 (8.8) | 60 (12.8) | 94 (12.8) | 0.762 |
| Age 60-80 | 466 (16.8) | 136 (18.1) | 27 (23.9) | 115 (15.8) | 188 (15.9) | 0.106 |
| Age ≥80 | 316 (24.0) | 109 (21.6) | 37 (49.3) | 104 (23.9) | 66 (22.1) | < 0.001 |
| 1-year mortality, n (%) | 1842 (31.4) | 609 (34.5) | 108 (44.1) | 490 (30) | 635 (28.6) | < 0.001 |
| Age < 60 | 380 (21.4) | 110 (21.5) | 10 (17.5) | 94 (20) | 166 (22.5) | 0.657 |
| Age 60-80 | 872 (31.5) | 261 (34.8) | 45 (39.8) | 222 (30.6) | 344 (29.1) | 0.014 |
| Age ≥80 | 590 (44.9) | 238 (47.1) | 53 (70.7) | 174 (39.9) | 125 (41.8) | < 0.001 |

**Supplement table S4. Multivariable Logistic analysis**

| **Variable** | **Total** | **Event %** | **Crude. OR ( 95 CI%)** | **Crude. *P* value** | **Adjusted.OR (95 CI%)** | **Adjusted. *P* value** |
| --- | --- | --- | --- | --- | --- | --- |
| BMI | 14883 | 2279 (15.3) | 0.98 (0.98~0.99) | < 0.001 | 0.98 (0.98~0.99) | < 0.001 |
| Healthy weight  (18.5–24.9 kg/m²) | 4208 | 740 (17.6) | 1(Ref) |  | 1(Ref) |  |
| Underweight  (<18.5 kg/m²) | 431 | 110 (25.5) | 1.61 (1.28~2.02) | <0.001 | 1.42 (1.09~1.84) | 0.008 |
| Overweight  (25.0–29.9 kg/m²) | 4908 | 692 (14.1) | 0.77 (0.69~0.86) | <0.001 | 0.78 (0.69~0.88) | <0.001 |
| Obese (≥30.0 kg/m²) | 5336 | 737 (13.8) | 0.75 (0.67~0.84) | <0.001 | 0.78 (0.69~0.89) | <0.001 |
| Trend.test | 14883 | 2279 (15.3) | 0.9 (0.86~0.93) | <0.001 | 0.91 (0.87~0.95) | <0.001 |

Adjusted for age, gender, race, SAPS, SOFA, Charlson Comorbidity Index, diabetes, hypertension, coronary artery disease, congestive heart failure, atrial fibrillation, malignancy cancer, stroke, chronic obstructive pulmonary disease, renal disease, liver disease and glucose.

**Supplement table S5. Univariate Logistic analysis**

|  | Statistics | 28-day mortality OR (95CI%) *P* value |
| --- | --- | --- |
| Age | 64.66 ± 15.55 | 1.02 (1.02, 1.03) < 0.0001 |
| Gender |  |  |
| Female | 5861 (39.38%) | 1 (Ref) |
| Male | 9022 (60.62%) | 0.80 (0.73, 0.88) < 0.0001 |
| BMI | 28.98 ± 7.53 | 0.98 (0.98, 0.99) < 0.0001 |
| SAPS | 39.16 ± 14.16 | 1.06 (1.05, 1.06) < 0.0001 |
| SOFA | 2.0 (0.0, 4.0) | 1.08 (1.06~1.1) < 0.001 |
| Charlson Comorbidity Index | 5.0 (3.0, 7.0) | 1.24 (1.22~1.26) < 0.001 |
| Hypertension |  |  |
| NO | 8136 (54.67%) | 1(Ref) |
| YES | 6747 (45.33%) | 0.72 (0.66, 0.79) < 0.0001 |
| Diabetes |  |  |
| NO | 10297 (69.19%) | 1(Ref) |
| YES | 4586 (30.81%) | 0.95 (0.86, 1.05) 0.2950 |
| Coronary artery disease |  |  |
| NO | 13215 (88.79%) | 1(Ref) |
| YES | 1668 (11.21%) | 1.09 (0.95, 1.25) 0.2316 |
| Congestive heart failure |  |  |
| NO | 12337 (82.89%) | 1(Ref) |
| YES | 2546 (17.11%) | 1.16 (1.03, 1.30) 0.0130 |
| Atrial fibrillation |  |  |
| NO | 9883 (66.40%) | 1(Ref) |
| YES | 5000 (33.60%) | 1.32 (1.21, 1.45) < 0.0001 |
| Chronic obstructive pulmonary disease |  |  |
| NO | 13849 (93.05%) | 1(Ref) |
| YES | 1034 (6.95%) | 1.76 (1.51, 2.05) < 0.0001 |
| Renal disease |  |  |
| NO | 2690 (18.07%) | 1(Ref) |
| YES | 12193 (81.93%) | 4.65 (3.85, 5.62) <0.0001 |
| Liver disease |  |  |
| NO | 11458 (76.99%) | 1(Ref) |
| YES | 3425 (23.01%) | 1.94 (1.77, 2.14) < 0.0001 |
| Stroke |  |  |
| NO | 13341 (89.64%) | 1(Ref) |
| YES | 1542 (10.36%) | 2.02 (1.78, 2.29) < 0.0001 |
| Malignancy cancer |  |  |
| NO | 11465 (77.03%) | 1(Ref) |
| YES | 3418 (22.97%) | 1.56 (1.42, 1.73) < 0.0001 |
| MAP (mm Hg) | 52.76 ± 11.26 | 0.93 (0.93, 0.93) < 0.0001 |
| Heart rate | 117.24 ± 24.42 | 1.02 (1.02, 1.02) < 0.0001 |
| PO2 | 73.73 ± 43.13 | 0.98 (0.98, 0.98) < 0.0001 |
| Lactate | 3.33 ± 2.85 | 1.24 (1.22, 1.26) < 0.0001 |
| Hemoglobin | 8.46 ± 1.86 | 0.91 (0.89, 0.93) < 0.0001 |
| pH | 7.29 ± 0.11 | 0.00 (0.00, 0.00) < 0.0001 |
| Creatinine | 1.56 ± 1.61 | 1.17 (1.14, 1.19) < 0.0001 |
| White blood cell counts | 18.16 ± 11.84 | 1.03 (1.02, 1.03) < 0.0001 |
| Mechanical ventilation use |  |  |
| NO | 1034 (6.95%) | 1(Ref) |
| YES | 13849 (93.05%) | 2.32 (1.83, 2.93) < 0.0001 |
| Vasopressor use |  |  |
| NO | 5977 (40.16%) | 1(Ref) |
| YES | 8906 (59.84%) | 1.85 (1.68, 2.04) < 0.0001 |
| Sedative use |  |  |
| NO | 13826 (92.90%) | 1(Ref) |
| YES | 1057 (7.10%) | 0.80 (0.66, 0.96) 0.0175 |

**Supplement table S6. Subgroup analysis**

| **Variable** | **Total** | **Event %** | **Crude.OR ( 95CI%)** | **Crude.*P* value** | **Adjusted.OR (95CI%)** | **Adjusted. *P* value** | *P* for interaction |
| --- | --- | --- | --- | --- | --- | --- | --- |
| **BMI (Female)** |  |  |  |  |  |  | 0.014 |
| Healthy weight  (18.5–24.9 kg/m²) | 1767 | 304 (17.2) | 1(Ref) |  | 1(Ref) |  |  |
| Underweight  (<18.5 kg/m²) | 245 | 69 (28.2) | 1.89 (1.39~2.56) | < 0.001 | 1.8 (1.28~2.53) | 0.001 |  |
| Overweight  (25.0–29.9 kg/m²) | 1632 | 279 (17.1) | 0.99 (0.83~1.19) | 0.933 | 0.99 (0.81~1.2) | 0.894 |  |
| Obese (≥30.0 kg/m²) | 2217 | 348 (15.7) | 0.9 (0.76~1.06) | 0.202 | 0.92 (0.76~1.11) | 0.386 |  |
| Trend.test | 5861 | 1000 (17.1) | 0.95 (0.9~1.01) | 0.082 | 0.96 (0.91~1.03) | 0.253 |  |
| **BMI (Male)** |  |  |  |  |  |  |  |
| Healthy weight  (18.5–24.9 kg/m²) | 2441 | 436 (17.9) | 1(Ref) |  | 1(Ref) |  |  |
| Underweight  (<18.5 kg/m²) | 186 | 41 (22) | 1.3 (0.91~1.87) | 0.155 | 1.04 (0.7~1.56) | 0.842 |  |
| Overweight  (25.0–29.9 kg/m²) | 3276 | 413 (12.6) | 0.66 (0.57~0.77) | < 0.001 | 0.67 (0.57~0.79) | < 0.001 |  |
| Obese (≥30.0 kg/m²) | 3119 | 389 (12.5) | 0.66 (0.56~0.76) | < 0.001 | 0.67 (0.57~0.8) | < 0.001 |  |
| Trend.test | 9022 | 1279 (14.2) | 0.86 (0.81~0.9) | < 0.001 | 0.86 (0.82~0.91) | < 0.001 |  |

Adjusted for age, gender, race, SAPS, SOFA, Charlson Comorbidity Index, diabetes, hypertension, coronary artery disease, congestive heart failure, atrial fibrillation, malignancy cancer, stroke, chronic obstructive pulmonary disease, renal disease,liver disease and glucose.


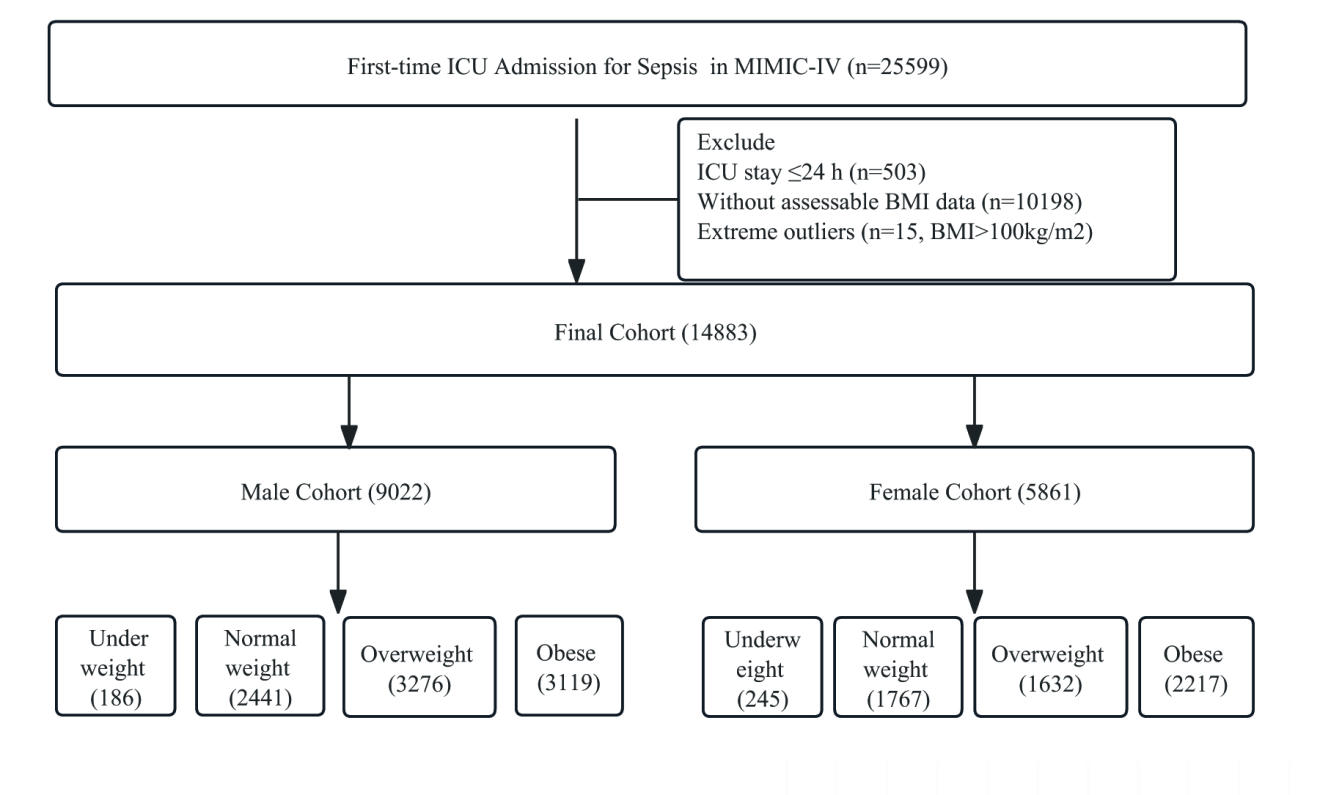


**Supplemental Figure S1**. Flowchart of patient selection. Illustration of exclusion and inclusion criteria in the process of selecting the final cohort of 14883patients.


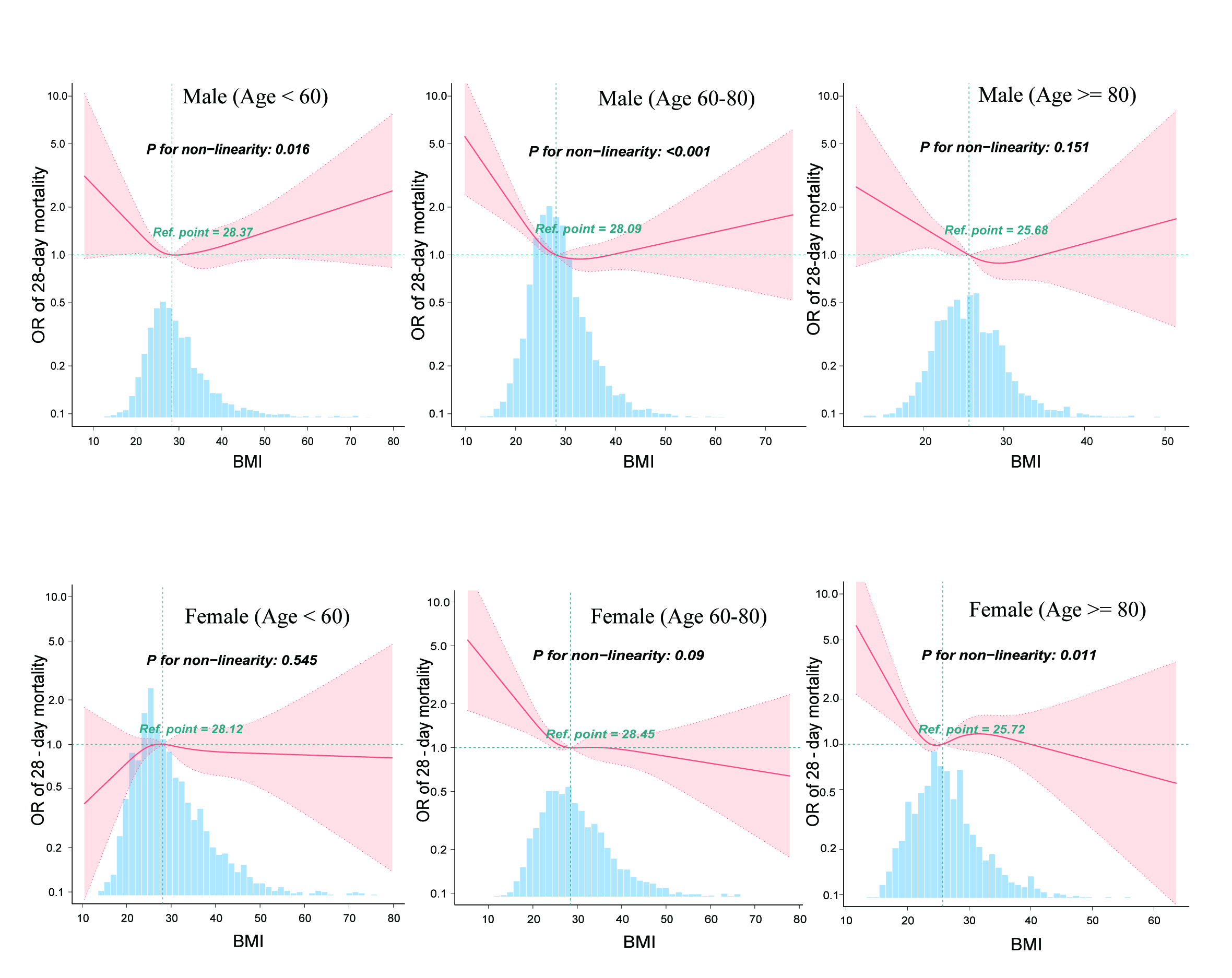


**Supplemental** Figure S2: Analysis of the correlation between BMI and 28-day mortality. The RCS model reveals a nonlinear correlation between BMI and 28-day mortality across various age groups, considering confounding factors such as gender, SAPS, SOFA, Charlson Comorbidity Index, diabetes, hypertension, coronary artery disease, congestive heart failure, atrial fibrillation, malignancy cancer, stroke, chronic obstructive pulmonary disease, renal disease and liver disease. The reference point for the Restricted Cubic Splines (RCS) is set to the median of the data, as indicated by the rcs.ref parameter. Abbreviations: OR (odds ratio).


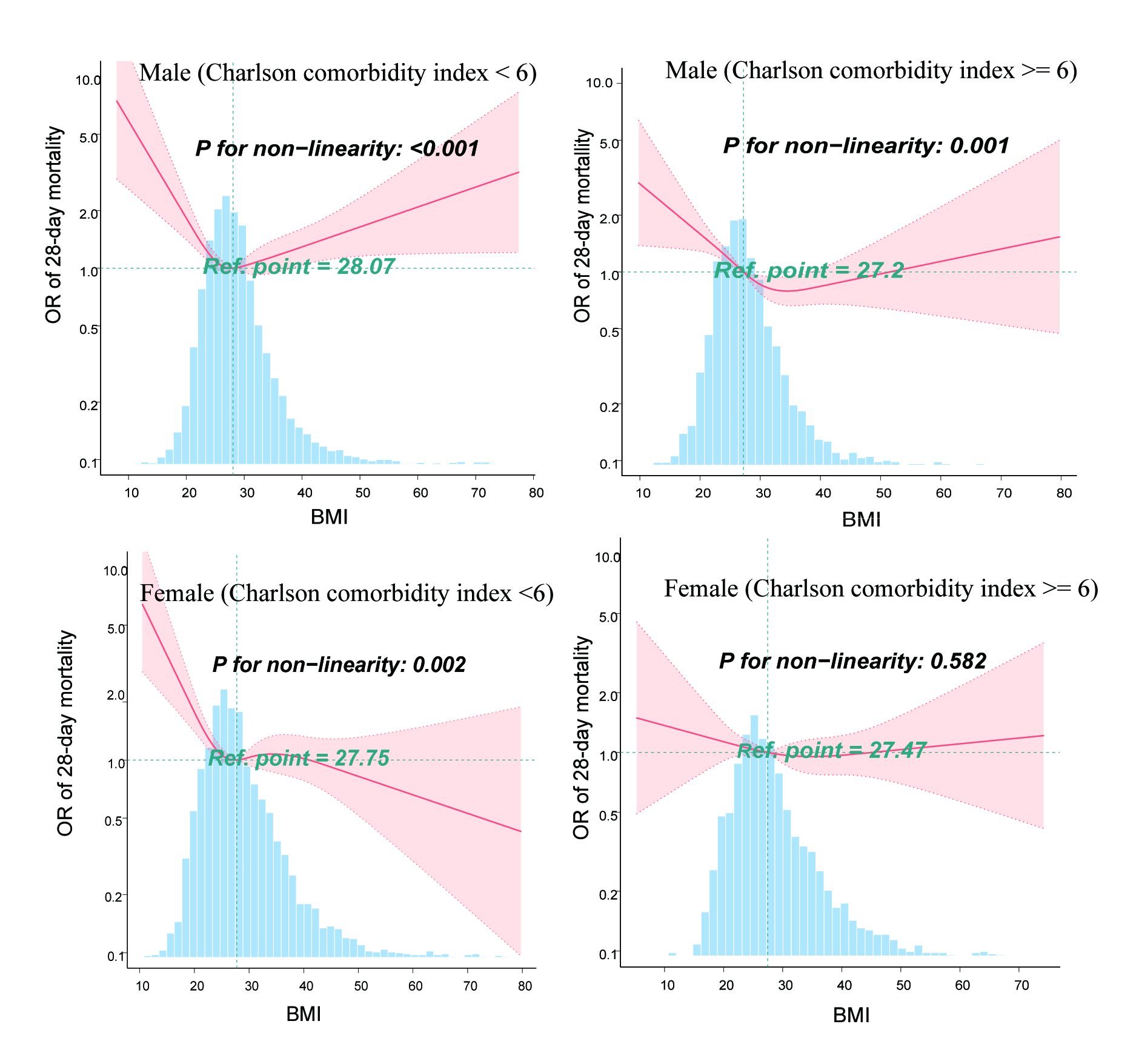


**Supplemental** Figure S3: Analysis of the correlation between BMI and 28-day mortality. The RCS model reveals a nonlinear correlation between BMI and 28-day mortality across various Charlson Comorbidity Index groups, considering confounding factors such as age, gender, diabetes, hypertension, coronary artery disease, congestive heart failure, atrial fibrillation, malignancy cancer, stroke, chronic obstructive pulmonary disease, renal disease and liver disease. The reference point for the Restricted Cubic Splines (RCS) is set to the median of the data, as indicated by the rcs.ref parameter. Abbreviations: OR (odds ratio).


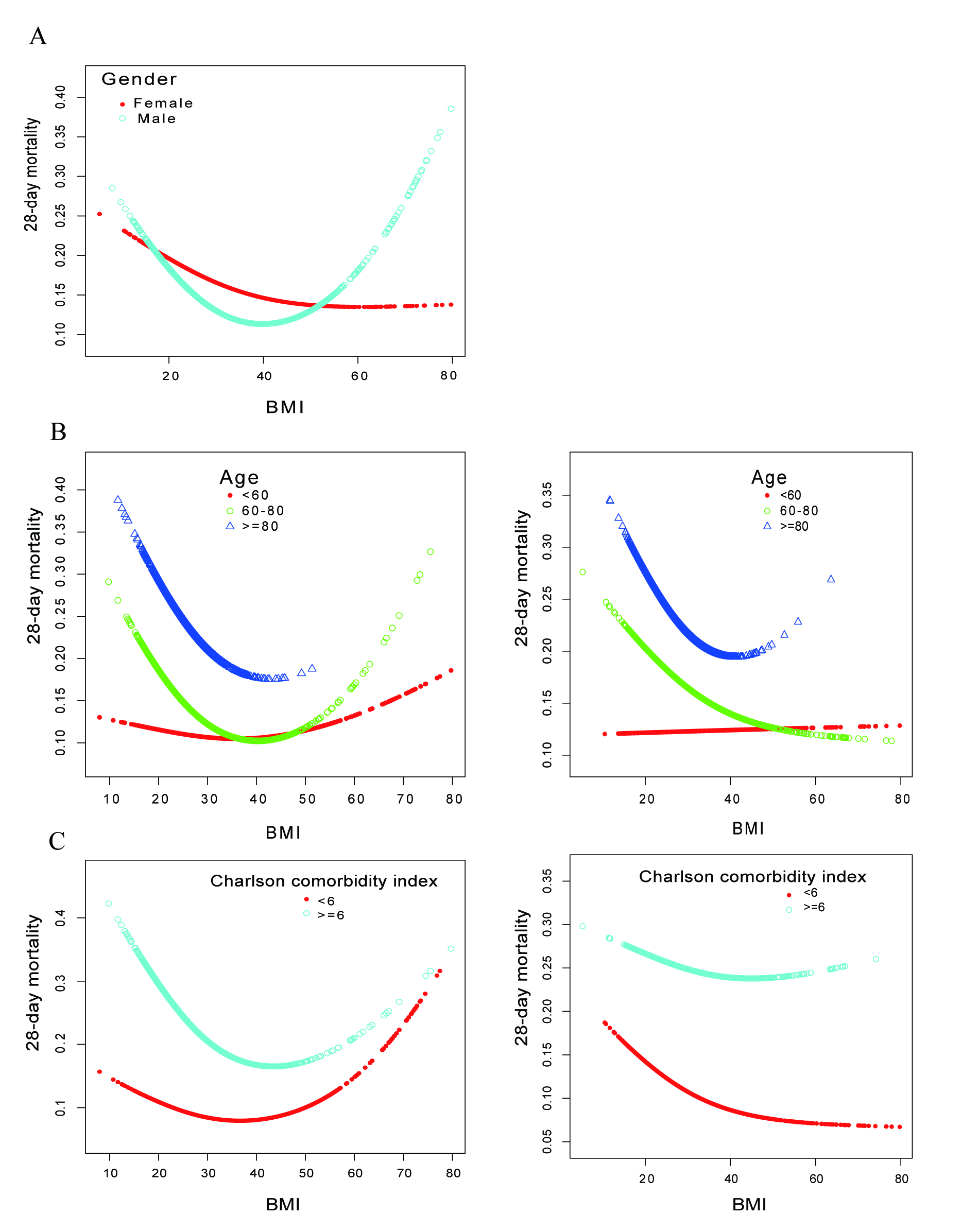


**Supplemental** Figure S4: The smoothed curve fitting graph illustrates the relationship between BMI and the 28-day mortality rate in sepsis patients.The smoothed curve fitting graph depicts the relationship between BMI and the 28-day mortality rate across different populations: (A) various genders, (B) different age groups, and (C) distinct Charlson Comorbidity Index groups.
